# Ontology for the Avida digital evolution platform

**DOI:** 10.1038/s41597-023-02514-3

**Published:** 2023-09-09

**Authors:** Raúl Ortega, Enrique Wulff, Miguel A. Fortuna

**Affiliations:** 1 Computational Biology Lab, Estación Biológica de Doñana (EBD), Spanish National Research Council (CSIC), Seville, Spain; 2 Instituto de Ciencias Marinas de Andalucía (ICMAN), Spanish National Research Council (CSIC), Puerto Real, Cádiz, Spain

**Keywords:** Computational platforms and environments, Software

## Abstract

The Ontology for Avida (OntoAvida) aims to develop an integrated vocabulary for the description of Avida, the most widely used computational approach for performing experimental evolution using digital organisms–self-replicating computer programs that evolve within a user-defined computational environment. The lack of a clearly defined vocabulary makes some biologists feel reluctant to embrace the field of digital evolution. This integrated framework empowers biologists by equipping them with the necessary tools to explore and analyze the field of digital evolution more effectively. By leveraging the vocabulary of Avida, researchers can gain deeper insights into the evolutionary processes and dynamics of digital organisms. In addition, OntoAvida allows researchers to make inference based on certain rules and constraints, facilitate the reproducibility of *in silico* evolution experiments and trace the provenance of the data stored in avidaDB–an RDF database containing the genomes, transcriptomes, and phenotypes of more than a million digital organisms. OntoAvida is part of the Open Biological and Biomedical Ontologies (OBO Foundry) and is available at http://www.obofoundry.org/ontology/ontoavida.html.

## Introduction

Since the pioneering work by Thomas S. Ray^1^ 30 years ago, digital evolution research has established itself as a valuable approach in biology, bridging experimental research with computational modelling. The contribution of digital evolution to the development of ecology and evolutionary biology comprises diverse topics such as robustness and evolvability^2–6^, complexity^1,7–10^, phenotypic plasticity^11,12^, the role historical contingency in evolution^13–15^, ecological interactions among species^16–21^, gene regulatory networks^2,22,23^, genomic architecture^9,24–26^, evolution of sex^27^, and evolution of cooperation^28–30^.

Avida is the most widely used software platform for research in digital evolution^31^. In Avida, self-replicating computer programs–digital organisms–evolve within a user-defined computational environment. It satisfies three essential requirements for evolution to occur: replication, heritable variation, and differential fitness. The latter arises through competition for the limited resources of memory space and central processing unit, CPU time. A digital organism in Avida consists of a sequence of code instructions–its genome–and a virtual CPU, which executes these instructions. Some of these instructions are involved in copying an organism’s genome, which is the only way the organism can pass its genetic material to future generations. To reproduce, a digital organism must copy this genome instruction by instruction into a new region of memory through a process that may lead to errors, i.e., mutations. A mutation occurs when an instruction is copied incorrectly, and is instead replaced in the offspring genome by an instruction chosen at random (with a uniform distribution) from a set of possible instructions. Some instructions are required for viability–replication–whereas others are required to complete computational operations (such as addition, multiplications, and bit-shifts), and are executed on binary numbers taken from the environment through input-output instructions encoded in the genome of the digital organism. The output of processing these numbers may equal the result of a specific Boolean logic operation, such as *NOT* and *NAND*. We call the identity of the logic operations it can perform the organism’s phenotype. An organism can be rewarded for performing logic operations with virtual CPU-cycles, which speeds up the execution of its instructions. This creates an additional selective pressure (besides reducing the number of instructions required for replication) which favours those organisms in an evolving population where mutations have produced sequences of instructions in their genomes that perform logic operations. Organisms that are more successful–those that replicate faster–are more likely to spread through a population.

Digital evolution presents a highly promising avenue within the field of ecology and evolution, offering an intermediary level of complexity between real-life systems and traditional mathematical models. By studying digital organisms, researchers gain unprecedented insights into evolutionary processes that are otherwise challenging to explore using natural systems alone. Consequently, digital organisms offer a complementary approach to studying natural or experimental evolution. In spite of the differences in the underlying mechanisms between digital and biological organisms, the fundamental operational processes, such as Darwinian evolution, remain equivalent.

Natural language has been used to describe the meaning of the data resulting from performing evolution experiments using Avida. In some cases, researchers have used different terms to refer to the same entity (e.g., avidian and digital organism, or functional trait and logic operation) or the same term to refer to different entities (e.g., genome and genotype, or phenotype and logic operation). The lack of a clearly defined vocabulary, including imprecision and ambiguity, makes some biologists feel reluctant to embrace the field of digital evolution. The first step in formalizing this knowledge is to define an explicit ontology that describes unambiguously the entities relevant to this particular domain and the relations between them. In the field of digital evolution, particularly in Avida, no effort has been made yet to formalize a vocabulary beyond the documentation provided with the software.

Developing an ontology for Avida can provide several benefits and advantages for studying and understanding digital evolution. Specifically, this standardized language can: enhance communication among researchers; improve our understanding of digital organisms (e.g., by defining the relationship between genotype and phenotype); help in formulating well-defined research questions, hypotheses, and data collection strategies; allow for better interoperability with other tools and platforms (e.g., aevol); be a valuable educational resource for those learning about digital evolution, artificial life, and evolutionary computation; provide a stable reference for understanding past experiments and their outcomes as the software and datasets evolve; enable effective data sharing and analysis within the research community (e.g., it allows researchers to compare results across different experiments); and finally, help identify areas for improvement and extension, supporting the continued growth of the software.

The Ontology for Avida (OntoAvida) is open and available to everyone and provides semantics to avidaDB–a database that stores genomes (i.e., circular sequences of code instructions), transcriptomes (i.e., the code instructions that are actually executed by the CPU of a digital organism during its replication), and phenotypes (i.e., logic operations computed on binary numbers taken from the environment) of more than a million digital organisms. The semantic relationships between the terms commonly used in Avida are expressed in the W3C standard ontology language OWL-DL. OntoAvida is part of the Open Biological and Biomedical Ontologies (OBO Foundry) and is currently developed by the Computational Biology Lab at the Doñana Biological Station, a research institute of the Spanish National Research Council based at Seville (Spain).

## Results

### Classes

**Fig. 1:**
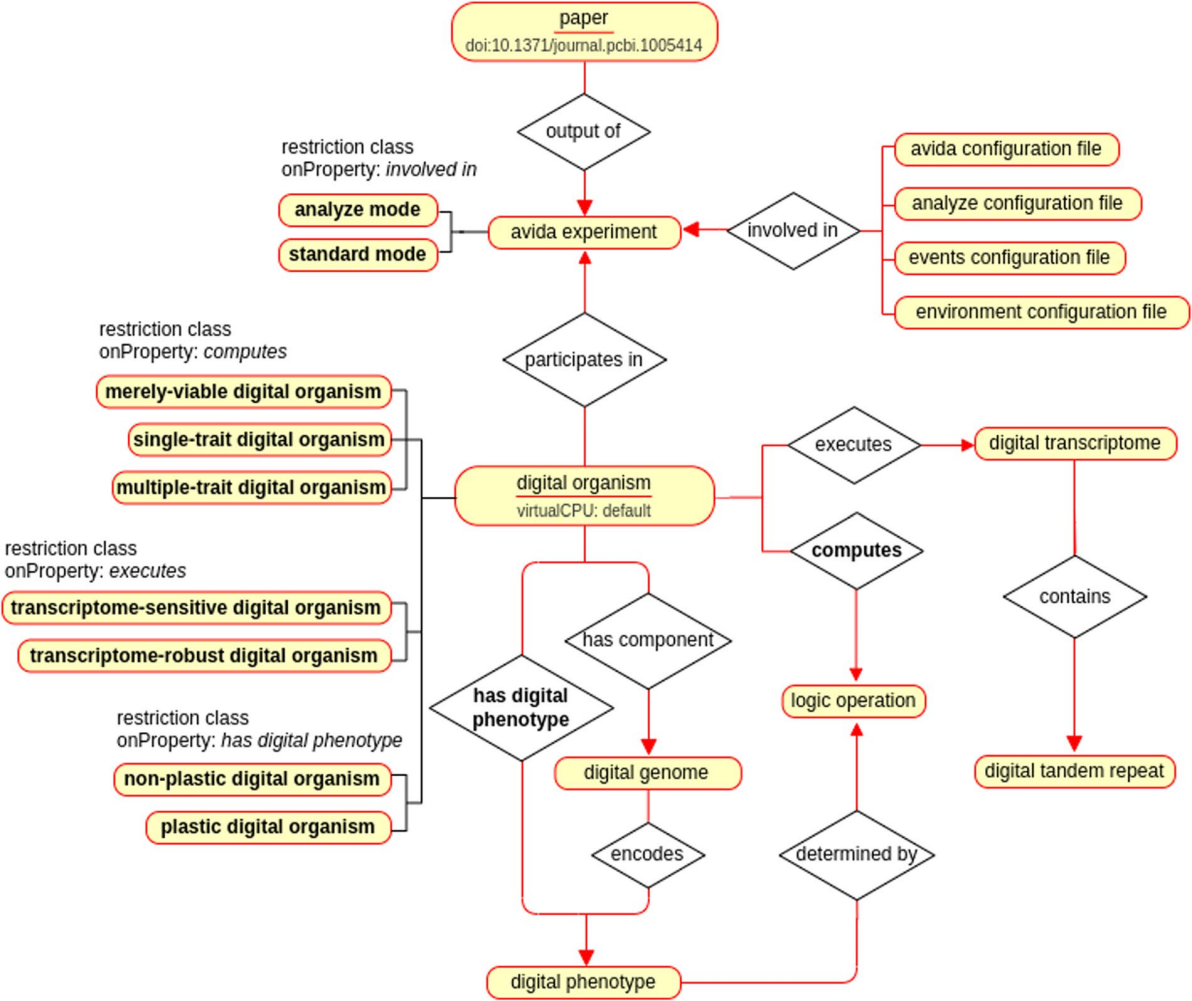
Subset of the ontology showing inference, provenance, and reproducibility. Classes and object properties are represented by rectangle and diamond symbols, respectively. The name of the inferred classes and properties are highlighted in bold face. Red arrows link classes connected by object properties, and black lines connect classes with their subclasses. Subclasses of the digital organism class are inferred based on restrictions applied on the properties computes, executes, and has phenotype (e.g., a merely-viable digital organism is a digital organism that does not compute any logic operation). Object properties are inferred as chain axioms (e.g., computes results from the combination of the properties has component, encodes, and determined by). We trace the provenance of the data stored in the database by indentifying the digital organisms that participate in an avida experiment reported in a scientific paper. In order to facilitate reproducibility in the results obtained from an avida experiment, we store the different configuration files associated to a particular experiment (e.g., analyze configuration file) as instances of their corresponding classes.

OntoAvida comprises 670 classes (without including imported terms). Some of the most relevant ones are the digital analogs of genome, transcriptome and phenotype. This set of terms includes the 512 subclasses corresponding to the distinct discrete phenotypes that can be computed by a digital organism (i.e., the whole phenotype space determined by the combination of 9 logic operations that can be computed by a digital organism). Next, we categorize the main classes following the three goals that have motivated the development of OntoAvida: inference, data provenance, and reproducibility (Fig. 1).

#### Inferrencing

Besides host and parasite digital organisms, 6 subclasses–organized in groups of disjoint classes–of the digital organism class are inferred from data:

- merely viable digital organism: a digital organism that does not compute any logic operation (i.e., it can only produce an offpring).
- single-trait digital organism: a digital organism that computes a single logic operation.
- plastic digital organism: a digital organism whose genome encodes distinct phenotypes in different environments.
- non-plastic digital organism: a digital organism whose genome encodes the same phenotype in all environments, respectively.
- transcriptome-robust digital organism: a digital organism that executes the same transcriptome in all environments.
- transcriptome-sensitive digital organism: a digital organism that executes distinct transcriptomes in different environments.

Inferrencing is performed using restrictions on the key object properties described in the next section: computes, executes, and encodes (used to infer the object property has phenotype).

#### Provenance

Provenance is an essencial requirement for building a semantic database because it contains information on the source from where the data stored in the database avidaDB come from. In OntoAvida, it involves three main classes:

- digital organism: a self-replicating computer program that mutates and evolves within a user-defined computational environment.
- avida experiment: an experiment carried out in Avida using digital organisms.
- paper as a specific class of publication: an article reporting original research, published in a peer-reviewed journal.

A digital organism can participate in or be formed as result of an avida experiment, depending on whether it already existed in the database or arised for the first time in a particular evolution experiment, respectively. In both cases, the experiment is reported and communicated to the scientific community in natural language mainly as a research paper, which is identified by the datatype doi (i.e., a character string used as a permanent identifier for a digital object, in a format controlled by the International DOI Foundation).

#### Reproducibility

When an evolution experiment is performed in Avida, anyone should be able to replicate the experiment and have access to the data.

### Object properties

A core set of 10 object properties have been added in the current version (without including imported terms). This set includes properties on ecological relations such as host-parasite interactions. Key properties such as computes, encodes and executes depend on the computational environment experienced by a digital organism (i.e., the seed used for starting the pseudo-random number generator). For example, the environment may modify the phenotype encoded by the genome of a digital organism every time an input-output instruction code is executed. Since this instruction code takes the content of the BX register of a digital organism and outputs it, checking it for any logic operations that may have been computed on the two 32-bit binary numbers stored in its input buffers, the phenotype of a digital organism depends on the content of the BX register when an input-output instruction code is executed, and this content depends initially–and later on, every time an input-output instruction code is executed–on the environment. Then, although the same genome could execute the same sequence of instruction codes in different environments, their BX registers might contain different binary numbers and hence, the input-output instruction code would output different binary numbers that might not be the result of computing the same logic operation. Therefore, the way the genome of a digital organism encodes a specific phenotype in a particular environment is implemented through the use of containers. A container is an RDF resource used to represent collections (https://www.w3.org/TR/rdf-schema/#ch_seq). Specifically we use the contained rdf:Seq because it preserves the order of each item stored in the container (e.g., rdf:_1, rdf:_2, …)–representing the environment or value of the seed–that are instances of the class rdfs:ContainerMembershipProperty (see an example of use by running a SPARQL query in the next section).

- computes: a relation between a digital organism and a logic operation, in which the digital organism performs the logic operation by executing the instruction codes harbored in its genome.
- encodes: a relation between the genome and the phenotype of a digital organism, in which the genome contains information that is used to produce the phenotype.
- executes: a relation between a digital organism and its transcriptome, in which the instruction codes harbored in its genome are executed to produce the transcriptome.
- mutant of: a relation between two digital organisms where their genomes differ in a single instruction code.

### Datatype properties

Only 16 datatype properties are included at this time (without including imported terms), but many more will be introduced in a near future. Since viable (i.e., the ability of a digital organism to produce an offspring able to replicate by executing its genome) and genome length executed (i.e., the number of instruction codes–out of the total number of instruction codes comprising a digital organism’s genome–that are executed by a digital organism during the replication process) might depend on the environment experienced by a digital organism (i.e., the seed used for starting the pseudo-random number generator), they are also implemented through the use of containers.

- genome instruction sequence: a genome instruction sequence is a linear string of letters representing the instruction codes that make up the genome of a digital organism.
- transcriptome instruction sequence: a transcriptome instruction sequence is a linear string of letters representing the instruction codes that make up the transcriptome executed by a digital organism.
- viable: the ability of a digital organism to produce an offspring able to replicate by executing its genome.

### Quering avidaDB

**Fig. 2:**
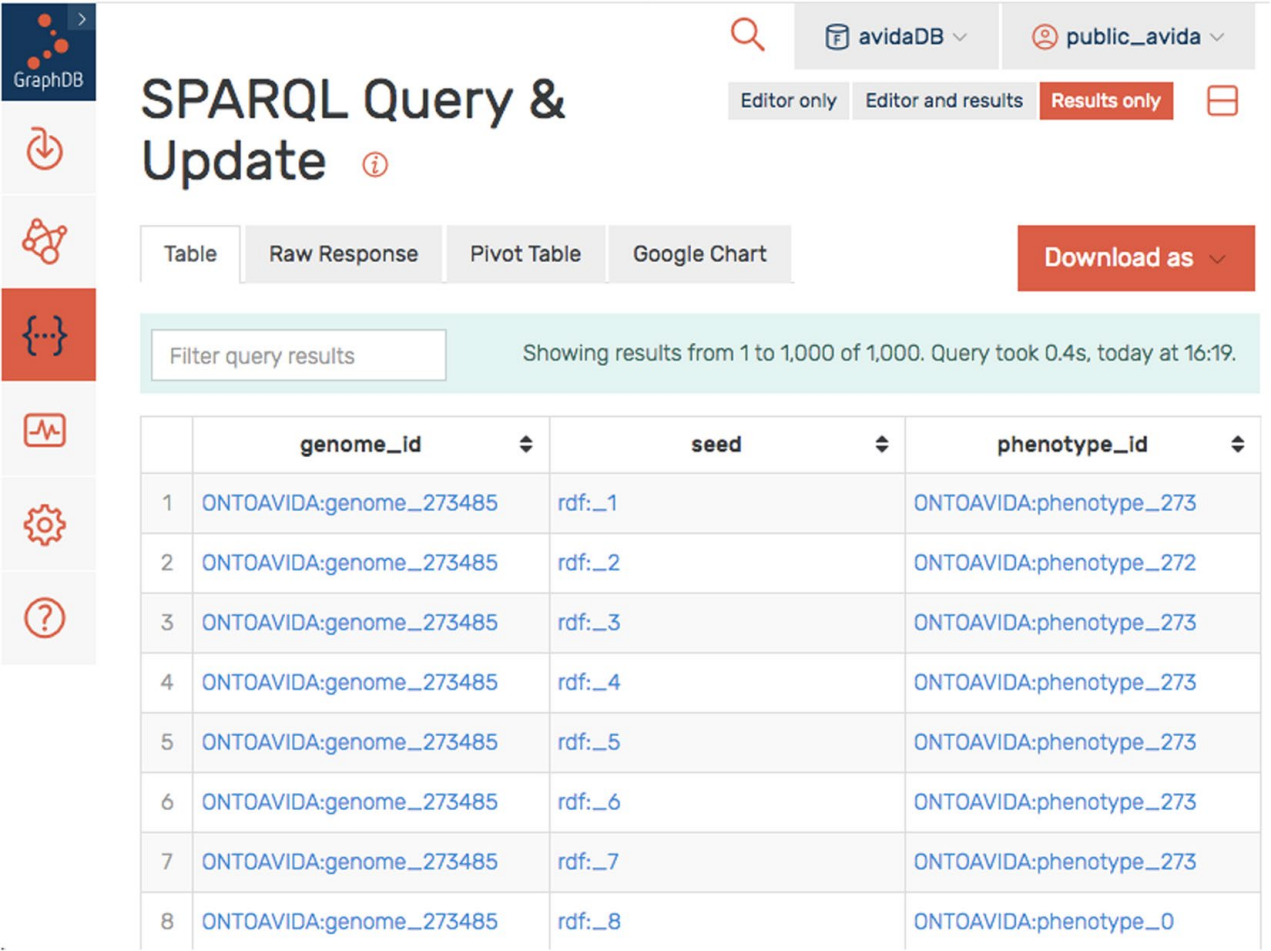
Ouput of the SPARQL query reported in the main text (first 8 lines). The SPARQL query retrieves, from avidaDB, the phenotypes encoded by the genome #273485 in 1000 distinct environments (from seed = 1 to seed = 1000). The predicate of the triples, seed, takes values rdf:_1, rdf:_2,…, which are instances of the class rdfs:ContainerMembershipProperty. That is, the value rdf:_1 points at the phenotype encoded by the genome at seed = 1. The first 8 lines of the table indicate that genome #273485 encodes phenotype #273 at seed = 1, seed = 3, seed = 4, seed = 5, seed = 6, and seed = 7, phenotype #272 at seed = 2, and phenotype #0 (i.e., merely-viable organisms) at seed = 8.

We take advantage of the phenotypic plasticity of digital organisms (i.e., the capability of the genome of a digital organism to encode different phenotypes in distinct computational environments) to illustrate the use of OntoAvida. We access the endpoint of avidaDB (https://graphdb.fortunalab.org) as anonymous user (i.e., username and password public_avida). The following SPARQL query retrieves the phenotypes encoded by the genome #273485 in 1000 distinct environments (see Fig. 2):

PREFIX ONTOAVIDA: http://purl.obolibrary.org/obo/ONTOAVIDA_

PREFIX rdfs: http://www.w3.org/2000/01/rdf-schema#

PREFIX rdf: http://www.w3.org/1999/02/22-rdf-syntax-ns#


SELECT?genome_id?seed?phenotype_idWHERE {?genome_id ONTOAVIDA:00001198?phenotype_seq.?phenotype_seq?seed?phenotype_id.?seed rdf:type rdfs:ContainerMembershipPropertyFILTER(?genome_id = ONTOAVIDA:genome_273485)}


The term ONTOAVIDA:00001198 (encodes seq) represents the relation between the genome of a digital organism and the container storing the phenotypes encoded by the genome in different environments (encodes at seed, being seed the integer used for starting the pseudo-random number generator). The object of the first triple of the WHERE clause (?phenotype_seq) is the container storing the phenotypes encoded by the genome of a digital organism in each environment. The triple?phenotype_seq?seed?phenotype_id obtains the members of the container, where?seed takes values rdf:_1, rdf:_2, …, and are instances of the class rdfs:ContainerMembershipProperty. The value rdf:_1 points at the phenotype encoded at seed = 1. For example, the first line of the output of this query, ONTOAVIDA:genome_273485 rdf:_1 ONTOAVIDA:phenotype_273, means that genome #273485 encodes phenotype #273 at seed = 1.

## Discussion

We have developed OntoAvida following the principles set by the Open Biological and Biomedical Ontologies (OBO) Foundry^32^, which are clearly aligned with the FAIR principles (i.e,. shared data should be Findable, Accessible, Interoperable, and Reproducible). Currently, OntoAvida is listed in the OBO registry (http://obofoundry.org) after passing the validation checks performed by the ROBOT software suite^33^.

OntoAvida is a valuable tool to perform studies that lie at the intersection of evolutionary biology and computer science. Its potential transcends traditional academic boundaries and will definitely provide fertile ground for new collaborations bridging these disciplines. Our ontology will appeal not only to students of evolutionary biology and computer science, but also to synthetic biologists, systems engineers, students of the origin of life, and philosophers.

## Methods

**Fig. 3:**
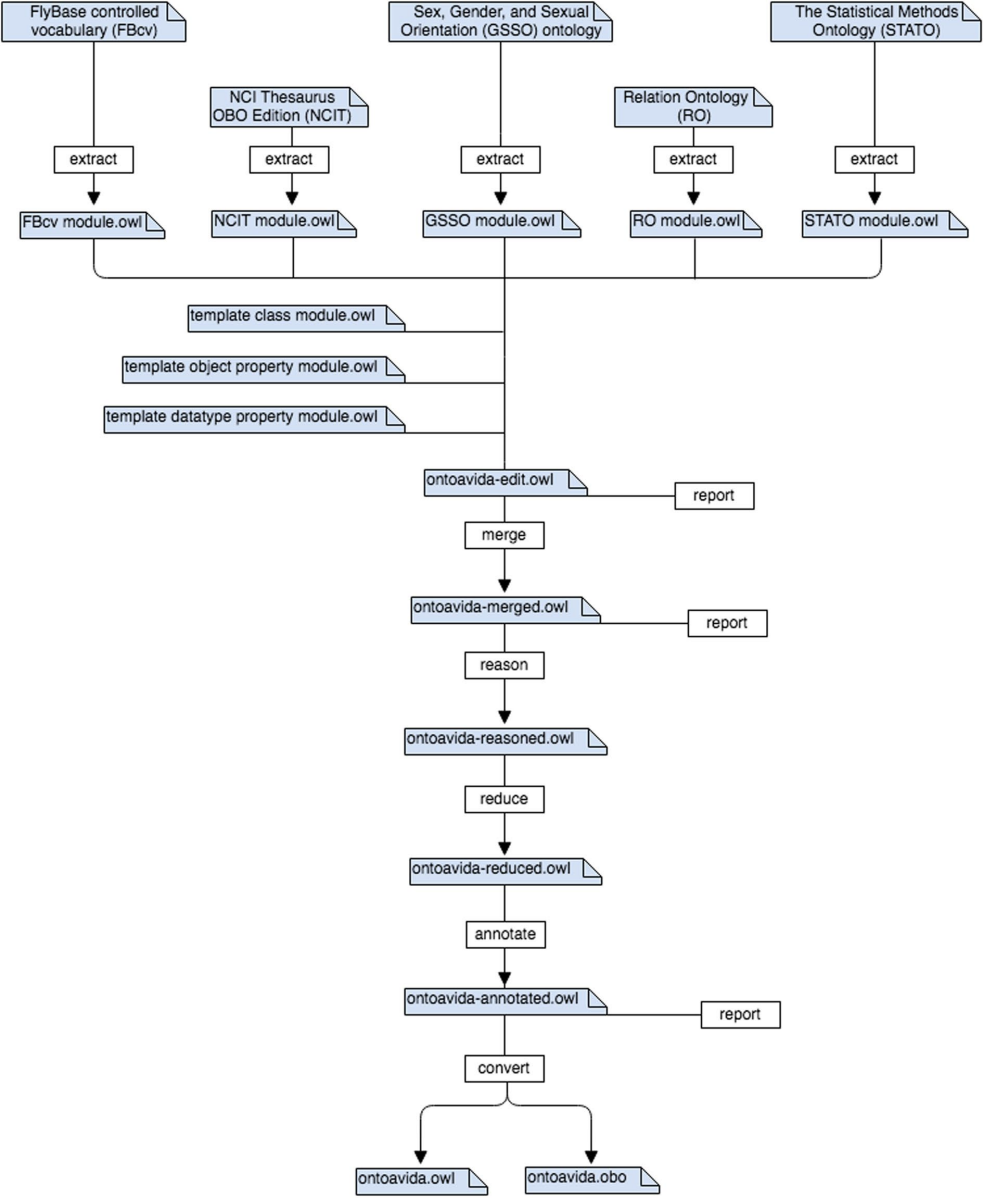
Ontology workflow. We automatized the development of the ontology using the following ROBOT pipeline (from top to bottom): selecting terms from external ontologies to reuse them (extract); integrating new terms proposed by contributors (template); merging imported and template modules with the core ontology (merge); checking the logical consistency of the ontology (reason, reduce, and annotate); and releasing the ontology (convert).

We used ROBOT commands^33^ to automatize the process of developing OntoAvida (Fig. 3). Next, we describe this process step by step.

### Selecting terms from external ontologies to reuse them

We identified the terms that can be reused from existing external ontologies, instead of creating new ones. When a term from our ontology was defined in more than one external ontology, we selected the ontology listed in the OBO registry because OntoAvida is intended to be part of an interoperable ecosystem with reuse of shared terms and relations^34^. We have reused classes from the following OBO ontologies: FlyBase controlled vocabulary (FBcv), Sex, Gender, and Sexual Orientation (GSSO) ontology, The Statistical Methods Ontology (STATO), and NCI Thesaurus OBO Edition (NCIT). All object properties reused from external ontologies come from the Relation Ontology (RO). Before extracting these terms and relations, we used a shell script that CURLs the external ontologies from their source URIs to download their latest versions. Then, we used the Syntactic Locality Module Extractor (SLME) algorithm from ROBOT to extract the terms as fix-point nested modules (STAR). We finally removed unwanted terms and annotations from the imported modules using remove and filter, and performed a quality control check on the extracted modules using report.

### Integrating new terms proposed by contributors

Contributors can propose new terms by adding them in template files (one for classes, another for object properties, and a third one for datatype properties). We converted the template files into OWL modules using template. Then, we annotated the modules using annotate, and performed a quality control check on the annotated template modules using report.

### Merging imported and template modules with the core ontology

The core OntoAvida ontology was built using Protégé^35^. We used merge to combine the imported modules, the template modules and the core ontology that contains the novel terms and relations edited in Protégé. We then performed a quality control check on the merged ontology using report.

### Checking the logical consistency of the ontology

We checked the logical consistency of the merged ontology using the following ROBOT commands: reason to perform an automatic classification of terms that involves asserting all inferred superclasses, relax to relax Equivalence Axioms to weaker SubClassOf axioms, and finally reduce to remove any redundant axioms introduced by the relax step. Then, we updated annotations before releasing the ontology (e.g., dated version IRI) using annotate, and validate the OWL-DL profile using validate-profile.

### Releasing the ontology

We used verify to create a list of terms (i.e., reporting the new terms added to the previous release of the ontology). We also converted the OWL annotated ontology to OBO (a format widely used in life science related ontologies) using convert. Finally, we renamed the latest version of the ontology as OWL and OBO files to stamp the date and placed them in the release folder of the GitLab repository .

**Fig. 4:**
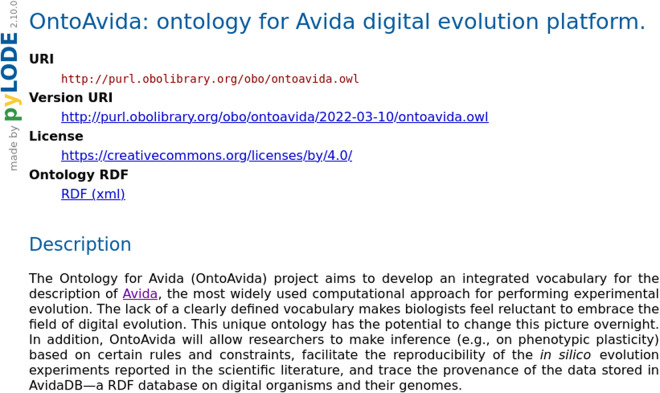
pyLODE visualization of OntoAvida. Screenshot of the HTML file generated for documenting the classes, object properties, and datatype properties of OntoAvida in an easy way.

## Data Availability

The endpoint of avidaDB (e.g., SPARQL GUI) can be accessed as anonymous user (i.e., username and password public_avida) at https://graphdb.fortunalab.org.

## Code availability

The ontology is available in both OBO and OWL format from the GitLab repository (https://gitlab.com/fortunalab/ontoavida) and can be found at https://gitlab.com/fortunalab/ontoavida/-/blob/master/ontoavida.obo and https://gitlab.com/fortunalab/ontoavida/-/blob/master/ontoavida.owl. OntoAvida OBO and OWL files are also available from the OBO Foundry (http://www.obofoundry.org/ontology/ontoavida.html). All files are available under the Creative Commons Attribution 4.0 International License (http://creativecommons.org/licenses/by/4.0/) which allows for the copying, redistribution and adaption of the ontology for any purpose.

pyLODE (https://github.com/rdflib/pyLODE) was used to obtain a user-friendly visualization of the ontology. pyLODE is based on the OWL Documentation Environment tool (LODE), implemented in Python, and used to generate human-readable HTML documents for OWL and RDF ontologies. We have customized the original pyLODE templates (https://gitlab.com/fortunalab/pyLODE) to convert a scheme of OntoAvida, in a HTML file so that its classes, object properties, and datatype properties can be easily visualized (Fig. 4). The pyLODE file of OntoAvida is available at https://owl.fortunalab.org/ontoavida.
